# Current Insights into Long Non-Coding RNAs (LncRNAs) in Prostate Cancer

**DOI:** 10.3390/ijms18020473

**Published:** 2017-02-22

**Authors:** Maria A. Smolle, Thomas Bauernhofer, Karl Pummer, George A. Calin, Martin Pichler

**Affiliations:** 1 Division of Clinical Oncology, Internal Medicine, Medical University of Graz, Auenbruggerplatz 15, A-8036 Graz, Austria; maria.smolle@cbmed.at (M.A.S.); thomas.bauernhofer@medunigraz.at (T.B.); 2 Department of Orthopaedic and Trauma Surgery, Medical University of Graz, Auenbruggerplatz 5, A-8036 Graz, Austria; 3 Department of Urology, Medical University of Graz, Auenbruggerplatz 5/6, A-8036 Graz, Austria; karl.pummer@medunigraz.at; 4 Department of Experimental Therapeutics, The University of Texas MD Anderson Cancer, 1515 Holcombe Blvd., Houston, TX 77030, USA; gcalin@mdanderson.org; 5 Center for RNA Interference and Non-Coding RNAs, The University of Texas MD Anderson Cancer Center, 1515 Holcombe Blvd., Houston, TX 77030, USA

**Keywords:** long non-coding RNAs, prostate cancer, carcinogenesis

## Abstract

The importance of long non-coding RNAs (lncRNAs) in the pathogenesis of various malignancies has been uncovered over the last few years. Their dysregulation often contributes to or is a result of tumour progression. In prostate cancer, the most common malignancy in men, lncRNAs can promote castration resistance, cell proliferation, invasion, and metastatic spread. Expression patterns of lncRNAs often change during tumour progression; their expression levels may constantly rise (e.g., HOX transcript antisense RNA, HOTAIR), or steadily decrease (e.g., downregulated RNA in cancer, DRAIC). In prostate cancer, lncRNAs likewise have diagnostic (e.g., prostate cancer antigen 3, PCA3), prognostic (e.g., second chromosome locus associated with prostate-1, SChLAP1), and predictive (e.g., metastasis-associated lung adenocarcinoma transcript-1, MALAT-1) functions. Considering their dynamic role in prostate cancer, lncRNAs may also serve as therapeutic targets, helping to prevent development of castration resistance, maintain stable disease, and prohibit metastatic spread.

## 1. Background

Prostate cancer is the most common malignancy in male and accounts for 13% of cancer-related deaths [1]. Established risk factors include race, advanced age and a family history of prostate cancer. X-linked, dominant and recessive inheritance is likewise suggested in family-associated prostate cancer [2]. From which component of the prostate malignant cells actually originate is controversial. Prostate cancer cells may derive from both basal and luminal epithelial cells. Another theory suggests that basal cells differentiate into glandular cells, thus forming the most frequent prostate cancer histological subtype, adenocarcinoma (prostate adenocarcinoma, PCA). The latter theory would be supported by the fact that one diagnostic feature of PCA is the lack of basal cells. However, based on results of molecular studies, the distinction between prostate cancer cells with ETS E-twenty-six fusion (ETS-positive) and cells lacking the fusion (ETS-negative) seems more adequate. Fusion of ETS family transcription members, for example the ETS-related gene (*ERG*) and ETS translocation variant 4 (ETV4) with the regulatory sequence of androgen-regulated genes such as transmembrane protease, serine 2 (*TMPRSS2*) brings transcription factors under androgen control [3]. Other genes frequently mutated in PCA include phosphatase and tensin homolog (*PTEN*), tumour protein p53 (*TP53*) and speckle-type POZ protein (*SPOP*) [4].

The androgen receptor (AR) is not only important for growth and differentiation of the healthy prostate, but also plays a crucial role in the pathogenesis of PCA [5]. It is expressed in nearly all primary prostate cancers [6]. The AR is usually bound to heat shock proteins (Hsp) such as Hsp90 in the cytoplasm [5]. After binding of either dihydrotestosterone (DHT) or testosterone to the AR, a conformational change results in release of the AR from Hsp90 [7]. The hormone-bound AR subsequently dimerises and translocates into the nucleus, where it regulates target gene expression [7,8]. Most tumours initially show response to androgen deprivation therapy (ADT), while remaining AR-positive and further responsive to AR-signalling [8]. Even more, some tumours develop mutations of the AR utilising hydroxyflutamide, a metabolite of the anti-androgen flutamide, as an agonist [9]. Moreover, tumours initiate expression of AR splice-variants that cannot be targeted by novel anti-androgens [10].

Treatment of localised PCA takes into account clinico-pathological factors including Gleason score, initial prostate-specific antigen (PSA) level, patient’s age and clinical tumour stage [11]. Active surveillance may be best for patients with low-risk disease (i.e., Gleason score of 6 or less), whereas men with high-risk disease (i.e., Gleason score >7, PSA levels >20 ng/mL and clinical tumour stage >pT2c, i.e., tumour involving both prostate lobes) will rather benefit from radical prostatectomy or radiotherapy [12].

The recommended therapy nowadays for locally advanced PCA consists of long-term ADT in combination with radiotherapy [13]. ADT decreases the circulating testosterone levels to a very low amount (<50 ng/mL), a condition called chemical castration of men. However, some tumours will become hormone refractory following ADT, featured by increasing PSA levels in blood and upregulation of the AR in cancer cells [14]. On the other hand, some patients present with primary metastatic PCA, creating a palliative treatment situation with the main goal to prolong survival time and to sustain quality of life. In this clinical setting, ADT is still the mainstay of therapeutic strategies. However, over a period of time of 12–36 months, a disease state called castration-resistant prostate cancer (mCRPC) evolves in almost every patient. The ineffectiveness of conventional ADT in these mCRPC is a result of androgen-independent activation of the AR and its downstream pathways [14]. Abiraterone and enzalutamide constitute potent novel AR-targeting drugs for patients with mCRPC. As a cytochrome P450 17A1 (CYP17A1) inhibitor, abiraterone impedes the production of androgens in adrenal glands, cancer cells and other sources [15], whilst enzalutamide directly binds to the AR and blocks nuclear translocation and activation of downstream AR-related pathways [16]. These novel anti-androgens have markedly improved life expectancy of patients with mCRPC [17]. However, tumours also develop resistance against these potent agents. As a consequence, taxane-based chemotherapy may be initiated or a bone-targeting agent (radium 231) might be administered [18].

The clinical course of PCA patients is variable. Some patients respond well to ADT for a period of many years, whilst others become refractory soon and show a dismal outcome. Ultimately, some of these mCRPCs may even transdifferentiate into a highly-aggressive neuroendocrine subtype. This is reflected in atypical metastatic spread to viscera and elevated levels of neuroendocrine markers such as neuron-specific enolase (NSE) and chromogranin A (CGA) [19].

## 2. Long Non-Coding RNAs

It has been long known that RNA exerts more functions than simply serving as a template for protein synthesis [20]. Whilst ribosomal (rRNA) and transfer RNA (tRNA) are relatively “old” discoveries, knowledge about presence and function of several other non-coding RNAs (ncRNAs) has evolved within the last few years [21]. Two main groups, namely small ncRNAs (sncRNAs) and long ncRNAs (lncRNAs) are distinguished by their molecular length, with a cut-off at 200 nucleotides [20,22]. Members of sncRNAs are microRNA (miRNA), small interfering RNA (siRNA), small nuclear RNA (snRNA) and the already mentioned tRNA.

LncRNAs are roughly divided into five categories according to their localisation on the genome. Sense lncRNAs overlap exons of another transcript on the same strand, whilst antisense lncRNAs are located on the opposite strand. Intergenic lncRNAs lie between two genes, bidirectional lncRNAs are transcribed simultaneously to coding transcripts at the opposite strand and intronic lncRNAs are located within the introns of a coding transcript [23,24]. However, recent evidence suggests that the latter group rather comprises fragments of unprocessed pre-mRNAs (messengerRNA) [25]. LncRNAs seem to be transcribed similarly to mRNAs, as both RNA polymerase II activity and histone modifications can be observed during transcription initiation and elongation of lncRNAs [26]. They can regulate gene expression directly in adjacent genomic loci (*cis*) or by initially interacting with transcriptional regulators (*trans*) [24].

LncRNAs are aberrantly expressed in a variety of human diseases, likewise contributing to pathogenesis or maintaining diseased conditions [27]. They exert their function via a multitude of mechanisms, including epigenetic silencing of gene clusters and transcriptional interference [24]. Due to their oncogenic or tumour-suppressive functions, various lncRNAs play an important role in the pathogenesis of many types of cancer including colorectal [28], kidney [29], breast [30], endometrial [31] and testicular cancer [32,33], as well as haematological types of cancer [34,35]. Aberrantly expressed lncRNAs can be indicative of certain stages of cancer progression, and may predict early progression or efficiently sustain tumour-related signalling pathways upon anti-cancer therapy [27].

## 3. Long Non-Coding RNAs and Prostate Cancer

**Figure 1:**
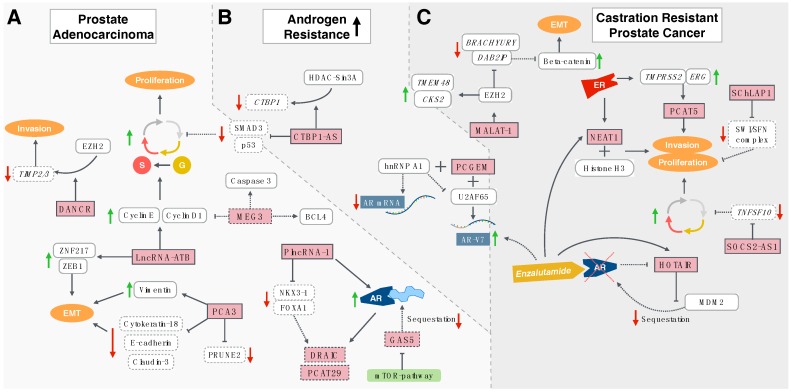
Molecular functions of long non-coding RNAs (lncRNAs) at different stages of prostate cancer, from prostate adenocarcinoma (**A**) to ongoing castration resistance (**B**) to a castration-resistant state (**C**). Dashed squares signify reduced or downregulated proteins/lncRNAs or genes, whilst solid boxes indicate overexpressed or upregulated proteins/lncRNAs or genes. Solid lines signify an active pathway, and dashed lines an inactive pathway. LncRNAs are framed by red, angular shaped boxes. Genes and proteins are framed by white boxes with blunt edges. DANCR: differentiation antagonising non-protein coding RNA; MEG3: lncRNA Maternally expressed gene 3; PCA3: prostate cancer antigen 3; DRAIC: Downregulated RNA in cancer; PCAT29: Prostate cancer-associated transcript 29; GAS5: growth arrest-specific 5; CTBP1-AS: C-terminal binding protein 1-antisense; PCGEM: prostate cancer gene expression marker 1; MALAT-1: metastasis-associated lung adenocarcinoma transcript-1; NEAT1: nuclear-enriched abundant transcript 1; PCAT5: prostate cancer-associated transcript 5; SChLAP1: Second chromosome locus associated with prostate-1; HOTAIR: HOX transcript antisense RNA; SOCS2-AS1: cytokine signalling 2-antisense transcript 1; TIMP 2/3: tissue inhibitor of metalloproteinase; EZH2: enhancer of zeste homolog; ZNF217: zink finger protein 217; ZEB1: zinc-finger E-box binding homeobox 1; PRUNE2: prune homolog 2; NKX3-1: homeobox protein Nkx-3.1; FOXA1: Forkhead box protein A1; BCL4: B-cell lymphoma like-2 like protein 4; SMAD3: Mothers against decapentaplegic homolog 3; CTBP1: C-terminal binding protein 1-antisense; HDAC-Sin3A: histone decarboxylase paired amphipathic helix protein Sin3a complex; TMEM48: transmembrane Protein 48; CKS2: cyclin-dependent kinases regulatory subunit 2; hnRNP A1: heterogeneous nuclear ribonucleoprotein A1; U2AF65: U2 Small Nuclear RNA Auxillary Factor 2; DAB2IP: disabled homolog 2-interacting protein; TMPRSS2: transmembrane Protease, Serine 2; ERG: ETS-(E-twenty-six) related gene; SWI/SFN complex: SWItch/Sucrose Non-Fermentable complex; TNSF10: tumour necrosis factor superfamily member 10; MDM2: mouse double minute 2 homolog.

The following review article will guide the reader through the disease process of prostate cancer. LncRNAs involved in the pathogenesis of hormone-sensitive PC (prostate cancer), those promoting castration resistance and lncRNAs mainly involved in mCRPC will be described (Figure 1).

Furthermore, their possible implication in clinical practice will be outlined (Table 1).

### 3.1. Hormone-Sensitive Prostate Cancer

#### 3.1.1. Long Non-Coding RNA Activated by Transforming Growth Factor-β 

Epithelial-to-mesenchymal transition (EMT) is an important factor promoting castration resistance of PC cells [36]. Cells undergoing EMT lose typical features of epithelial cells such as cell–cell adhesion and polarity. On the other hand, they gain functions of mesenchymal cells, including migratory, invasive and anti-apoptotic potential [36,37,38]. Androgen deprivation enhances EMT in PCA, reflected in elevated mesenchymal markers (e.g., N-cadherin, zinc finger protein SNAI2 (Slug), zinc-finger E-box binding homeobox 1 (ZEB1)) and decreased epithelial markers such as E-cadherin [39]. In PCA, the long non-coding RNA activated by transforming growth factor (TGF)-beta (lncRNA-ATB) induces expression of ZEB1 and zinc-finger protein 217 (ZNF217), that are both involved in EMT [40]. High lncRNA-ATB levels positively correlate with unfavourable clinical features including high Gleason-score, several lymph node metastases, elevated PSA, histological grade and anatomical stage [40]. This may in part be caused by the EMT-promoting effect of lncRNA-ATB, as EMT enables prostate cancer cells to migrate, invade and ultimately set metastases [41]. Additionally, lncRNA-ATB stimulates progression through G1-phase as well as the transition from G1 into S-phase of the cell cycle by upregulating cyclin D1 and cyclin E (Figure 1A) [40]. As a consequence, proliferation rate of prostate cancer cells is enhanced upon overexpression of lncRNA-ATB [40]. Therefore, lncRNA-ATB could serve as a prognostic biomarker in PCA and therapeutic blockage may decelerate tumour progression.

#### 3.1.2. Prostate Cancer Antigen 3

The lncRNA prostate cancer antigen 3 (PCA3) is one of the most specific PCA biomarkers, being significantly upregulated in cancer compared with healthy prostate [42]. In combination with other clinical measures such as PSA, patient’s age, prostate volume and findings on digital rectal examination, testing of PCA3 urine levels reliably predicts the chance of an underlying prostate cancer [43,44]. As a result, patients can be saved from an unnecessary diagnostic prostate biopsy in clinical practice by additionally taking into account urine PCA3 levels [45]. Moreover, low PCA3 scores predict insignificant and small-volume PCA [46]. However, PCA3 levels do not correlate with tumour grade or aggressiveness. Therefore, its use as a prognostic marker is limited [46,47].

The high clinical relevance notwithstanding, the function of PCA3 is not limited to one specific process. Rather, it is involved in several mechanisms, including regulation of cancer genes and AR cofactors as well as partial modulation of EMT [48].

The experimental knockdown of PCA3 leads to upregulation of epithelial markers E-cadherin, claudin-3, and cytokeratin-18, with consecutive downregulation of the mesenchymal marker vimentin [48]. Of note however, is that the EMT pattern is not completely reversed by PCA3 knockdown; mesenchymal markers zinc finger protein SNAI1 (Snail), Twist-related protein 1 (TWIST1) and Slug are still found upregulated and epithelial markers cytokeratin-8 and claudin-4 downregulated upon PCA3 knockdown [48].

Moreover, PCA3 regulates expression of important cancer genes involved in angiogenesis (vascular endothelial growth factor A (VEGFA), fibrillin-1 (IFNB1)), cell adhesion (metastasis suppressor protein 1 (MTSS1), integrin β-1 (ITGB1)), signal transduction (receptor tyrosine-protein kinase (ERBB2), phosphatidylinositol 3-kinase regulatory subunit α (PIK3R1)), mitogen-activated protein kinase kinase 1 (MAP2K1), and apoptosis (Bcl-2-associated death promoter (BAD), telomerase reverse transcriptase (TERT)) [48].

Being located in the intronic antisense region of prune homolog 2 (*PRUNE2*) gene, PCA3 also modulates expression of this tumour suppressor [49]. After formation of a nuclear PRUNE2/PCA3 double-stranded (ds) RNA, the adenosine deaminases acting on RNA (ADARs) bind to the dsRNA, promote adenosine-to-inosine editing and subsequently repress translation of the complex (Figure 1A) [49]. High PRUNE2 levels are associated with reduced prostate cancer cell proliferation, whilst silencing of this tumour suppressor enhances proliferation and transformation. Conversely, PCA3 underexpression diminishes and PCA3 overexpression induces prostate cancer cell proliferation [49]. In healthy prostate tissue, high PRUNE2 levels involve low PCA3 levels, this being the other way around in PC samples [49]. Besides its application as a diagnostic biomarker, PCA3 may in the future also serve as a therapeutic target to reduce progression of PCA.

#### 3.1.3. Maternally Expressed Gene 3 

The lncRNA Maternally expressed gene 3 (MEG3) is involved in cell cycle regulation. Expression levels are significantly lower in PCA as compared with the healthy prostate [50]. MEG3 normally acts as a tumour suppressor, as it likewise impairs cell proliferation and promotes apoptosis by activating p53 [51]. It enhances expression of caspase 3 as well as the pro-apoptotic Bcl-2 (B-cell lymphoma-like 2)-like protein 4 (BCL2L4) [52]. Moreover, MEG3 inhibits expression of cyclin D1 and B-cell lymphoma 2 (Bcl-2) at the post-transcriptional level, hence inducing cell cycle arrest [50]. Consequently, overexpression of MEG3 leads to cell cycle arrest and induction of apoptosis in PCA. Conversely, underexpression of MEG3 results in enhanced cell proliferation. Thus, low levels of MEG3 may contribute to tumourigenesis in PCA due to uninhibited expression of Bcl-2 and cyclin D1 (Figure 1A) [50]. Nevertheless, MEG3-levels do not seem to correlate with clinical features of PCA, including Gleason score, PSA levels and number of lymph node metastases [50]. By therapeutically inducing MEG3 expression in PCA, its tumour-suppressive effects may help to control progress of disease.

#### 3.1.4. Differentiation Antagonising Non-Protein Coding RNA 

In the healthy prostate, the androgen–AR signalling pathway drives terminal differentiation of epithelial cells [53]. In PCA, a functioning androgen–AR axis prevents invasion and metastasis of malignant cells [54]. At the same time, systemic deprivation of androgens is used for treatment of hormone-sensitive PCA. As aforementioned, most PCAs become resistant to conventional ADT by circumventing the androgen–AR interaction necessary for activation of downstream pathways. In such cases, enzalutamide can be used, as it both directly targets the AR and inhibits downstream signalling pathways. Paradoxically, enzalutamide also seems to induce PCA cell invasion and metastasis [55]. In this context, the differentiation antagonising non-protein coding RNA (DANCR) may play an important role. This lncRNA normally suppresses differentiation of epithelial cells and hence may counteract the effect exerted by the androgen-AR pathway in the prostate [56]. DANCR is frequently overexpressed in PC and leads to downregulation of the TIMP (Tissue inhibitor of metalloproteinase) metalloproteinase inhibitors 2 and 3 (TIMP2/3), that usually prevent invasion and metastatic spread [57]. Moreover, DANCR seems to mediate binding of the epigenetic gene silencer enhancer of zeste homolog (EZH2) to TIMP2/3, most likely by acting as a scaffold lncRNA (Figure 1A) [57]. On the other hand, the androgen-AR axis reduces DANCR levels whilst enhancing expression of TIMP2/3. In the case resistance against conventional anti-androgens develops and enzalutamide is administered to block the androgen–AR axis more efficiently, expression of DANCR is no longer suppressed, leading to a reduction of TIM2/3 levels and initiation of migration, invasion and metastasis [57]. At the same time, knockdown of DANCR reduces the proportion of migrating and invading prostate cancer cells upon treatment with enzalutamide. Therefore, the simultaneous blockage of DANCR during treatment with enzalutamide could potentially inhibit cellular migration and prevent metastatic spread [57].

#### 3.1.5. Prostate Cancer-Associated Transcript 29 and Downregulated RNA in Cancer 

Two more lncRNAs regulated by the androgen–AR-axis are the Downregulated RNA in cancer (DRAIC) and the Prostate cancer-associated transcript 29 (PCAT29) [58,59]. The PCAT29-locus is situated 20 kilobases downstream to DRAIC [58]. The AR is recruited both to the DRAIC and PCAT29 gene loci and represses their transcription. Conversely, the transcription factors forkhead box protein A1 (FOXA1) and homeobox protein Nkx-3.1 (NKX3-1) counteract the suppressive effect of AR on PCAT29 and DRAIC gene loci by inducing their transcription [58]. During the progression of hormone-sensitive PCA towards mCRPC, the combination of decreasing FOXA1 and NKX3-1 levels as well as an aberrantly activated androgen–AR axis result in decreased levels of DRAIC and PCAT29 (Figure 1A) [58]. Both lncRNAs usually exert suppressive functions by preventing migration and metastatic spread of prostate cancer cells. However, whilst PCAT29 inhibits proliferation, DRAIC seems to rather promote cellular division [58,59]. This contradiction again demonstrates the complex and widespread function of lncRNAs in cancer. Importantly, low levels of PCAT29 and DRAIC are associated with increased rates of biochemical recurrence in patients with localised PCA [58,59]. Therefore, patients with low PCAT29 and DRAIC levels may be followed-up more closely so as not to miss progression into a castration-resistant state.

#### 3.1.6. PlncRNA-1

PlncRNA-1 is another lncRNA interacting with the AR [60]. It is likewise overexpressed in androgen-sensitive (LnCaP) and androgen-resistant prostate cancer cell lines (LnCaP-AI (androgen insensitive), PC3) compared to healthy prostate and benign prostatic hyperplasia (BPH) [60]. 

Knockdown of PlncRNA-1 leads to enhanced apoptosis both in androgen-sensitive and androgen-resistant cell lines. Moreover, PlncRNA-1 expression levels positively correlate with AR mRNA levels. Interestingly, knockdown of PlncRNA-1 does not only diminish expression of AR mRNA and AR protein, but is also associated with reduced levels of NKX3-1, a downstream target of the AR (Figure 1A) [61]. Conversely, PlncRNA-1 levels decrease upon AR-knockdown, although the underlying mechanisms of this reciprocal regulation are still unknown [60]. Hence, blockage of this lncRNA may aid in decelerating prostate cancer progression.

#### 3.1.7. Growth Arrest-Specific 5 

The growth arrest-specific 5 (GAS5) lncRNA belongs to the 5′ terminal oligopyrimidine gene family. It is encoded by the *GAS5* gene on 1q25, a PCA-associated locus [62]. In proliferating cells, GAS5 translation is regulated by the mechanistic target of rapamycin (mTOR) pathway and the nonsense-mediated decay (NMD) pathway. The latter one degrades transcripts with stop codons in early exons and therefore also GAS5, which comprises a stop codon at exon 3 [63]. Active mTOR pathway promotes translation of the GAS5 short reading frame. As NMD subsequently degrades these GAS5 transcripts, GAS5 levels decrease. During growth arrest, activity of the mTOR pathway is suppressed and active translation with consecutive NMD-degradation of GAS5 transcripts is diminished. Hence, GAS5 transcripts accumulate within the resting cell [64].

In the context of PCA, GAS5 promotes cancer cell death and prevents binding of the androgen/AR-complex to target DNA by sequestering the complex [65]. GAS5 is downregulated in mCRPC and low levels of this lncRNA are associated with reduced chemotherapy-induced cellular apoptosis (Figure 1A) [66]. The use of mTOR-inhibitors leads to an increase of GAS5 in androgen-dependent and androgen-sensitive prostate cancer cell lines but not in androgen-independent cell lines [67]. Moreover, GAS5 is necessary for proper mTOR-inhibitor function. In clinical practice, mTOR-inhibitors may be used in early-stage PCA to enhance cellular apoptosis by up-regulating GAS5. On the other hand, the ineffectiveness of mTOR-inhibitors in castration-resistant cells is most likely related to low GAS5-levels [67].

### 3.2. Promoters of Castration Resistance

#### 3.2.1. Prostate Cancer Gene Expression Marker 1 

An important mechanism through which PC cells can circumvent ADT is by expressing AR splice variants, ultimately leading to castration resistance. Seven AR splice variants are known to date, with AR-V7 (Androgen splice-variant 7; AR3) having the highest clinical relevance [10,68]. The lncRNA prostate cancer gene expression marker 1 (PCGEM1) is elevated in up to 80% of PCA tissues and enhances proliferation whilst inhibiting apoptosis [69]. ADT leads to an up-regulation of PCGEM1, which is subsequently re-located into the nucleus [70]. Upon ADT, the interaction of PCGEM1 with the splicing factors U2 Small Nuclear RNA Auxillary Factor 2 (U2AF65) and heterogeneous nuclear ribonucleoprotein A1 (hnRNP) is enhanced [70]. Binding of PCGEM1 to hnRNP A1, a negative regulator of AR-V7, reduces the affinity of hnRNP A1 to AR pre-mRNA as well as its ability to suppress U2AF65 binding to the same pre-mRNA. In parallel, the interaction of PCGEM1 with U2AF65, an enhancer of AR-V7, leads to an increased activity of the splicing factor to AR pre-mRNA (Figure 1B) [70]. Consequently, the upregulation of PCGEM1 under androgen-deprived conditions leads to expression of AR-V7, thus promoting therapy resistance and development of mCRPC [70]. In clinical practice, simultaneous blockage of PCGEM1 upon ADT may enhance efficacy of therapeutic agents.

#### 3.2.2. C-Terminal Binding Protein 1-Antisense

The lncRNA C-terminal binding protein 1-antisense (CTBP1-AS) is located in the antisense region of the C-terminal binding protein 1 (*CTBP1*) gene and is frequently overexpressed in PCA [71]. It represses transcription of CTBP1 by recruiting histone decarboxylase (HDAC)-paired amphipathic helix protein Sin3a (Sin3A) complexes to the gene’s promoter region, after having associated with the transcriptional repressor PTB (Polypyrimidine Tract Binding Protein)-associating splicing factor (PSF) [71]. Thus, high levels of CTBP1-AS are accompanied by reduced CTBP1 levels.

Moreover, PSF and CTBP1-AS promote cell cycle progression by repressing Mothers against decapentaplegic homolog 3 (SMAD3) and p53, two cell cycle inhibitors usually regulated by the AR (Figure 1B) [71]. Additionally, CTBP1-AS itself mimics AR signalling by inducing upregulation of AR-regulated genes.

During ADT, expression levels of CTBP1-AS constantly increase. Especially in androgen-deprived conditions, the lncRNA promotes cell cycle progression and tumour cell proliferation [71]. During treatment with anti-androgens, tumour cell proliferation may be reduced via targeting CTBP1-AS and its protein partner PSF.

### 3.3. Castration-Resistant Prostate Cancer

#### 3.3.1. HOX Transcript Antisense RNA

The HOX transcript antisense RNA (HOTAIR) lncRNA is usually repressed by the AR. Consequently, HOTAIR is significantly overexpressed in mCRPC as compared with early-stage PCA [72]. By binding to the N-terminal domain (NTD) of the AR protein, HOTAIR prevents interaction of the mouse double minute 2 homolog (MDM2), an E3 ubiquitin ligase, with the NTD. Hence, ubiquitination and degradation of the AR is prevented (Figure 1C) [72]. Moreover, HOTAIR induces and maintains activation of the AR in an androgen-independent manner.

Overexpression of HOTAIR enhances proliferation and invasion of castration-resistant cells. Additionally, levels of HOTAIR constantly increase in LNCaP cell lines upon treatment with enzalutamide [72]. This could partially explain the clinical observation of resistance development during enzalutamide treatment. In clinical practice, HOTAIR may serve as a biomarker indicating resistance against enzalutamide. In addition, concurrent targeting of HOTAIR potentially enhances anti-proliferative effects exerted by novel anti-androgens such as enzalutamide [72].

#### 3.3.2. Metastasis-Associated Lung Adenocarcinoma Transcript-1 

As its name implies, the metastasis-associated lung adenocarcinoma transcript-1 (MALAT-1) was first identified as a prognostic factor in bronchial carcinoma [73]. In the prostate, expression levels of MALAT-1 dramatically increase during progress from hormone-sensitive to castration-resistant conditions [74]. MALAT-1 binds to EZH2, a core subunit of polycomb-repressive complex 2 (PRC2) that is also frequently overexpressed in PCA [75,76]. This interaction results in enhanced repression of such polycomb-dependent target genes such as the disabled homolog 2-interacting protein (DAB2IP) and BRACHYURY [75]. In PCA, the DAB2IP protein inhibits EMT by enhancing degradation of β-catenin, an inducer of EMT [77]. Moreover, the interaction of MALAT-1 with EZH2 also regulates polycomb-independent expression of genes, including transmembrane Protein 48 (TMEM48) and cyclin-dependent kinases regulatory subunit 2 (CKS2) (Figure 1C) [75]. Migration and invasion of castration-resistant cells is enhanced by MALAT-1, whilst knockdown of this lncRNA results in decreased cellular invasion and leads to de-repression of DAB2IP [75].

Clinically, MALAT-1 correlates with advanced tumour stage, elevated PSA levels and resistance to ADT [74]. Moreover, it may serve as a diagnostic non-invasive biomarker aiding detection of prostate adenocarcinoma; urine MALAT-1-levels predict the PCA-risk even more accurately than routine PSA screening and would possibly prevent one-third of unnecessary prostate biopsies [78].

#### 3.3.3. Nuclear-Enriched Abundant Transcript 1

In mCRPC, signalling through the oestrogen receptor α (ERα) constitutes an effective mechanism to bypass the androgen–AR axis [79]. Whilst the ERα is absent in healthy prostate epithelium, it is overexpressed in all types of PCA. It regulates important steps in oncogenesis, such as the expression of the Transmembrane Protease, Serine 2 (TMPRSS2) -ERG fusion gene, which is frequently found in ETS-positive PCA types [80]. Moreover, ERα induces transcription of the lncRNA nuclear-enriched abundant transcript 1 (NEAT1) in PCA [79].

NEAT1 is recruited to the promoter regions of specific PCA genes. It epigenetically induces an environment in favour of active transcription by binding to histone H3 [79]. Moreover, NEAT1 promotes cellular proliferation and invasion, both in vitro and in vivo (Figure 1C) [79].

Notably, NEAT1 levels increase upon long-term treatment with either tamoxifen, bicalutamide or enzalutamide [79]. Accordingly, both ERα and NEAT1 levels are significantly higher in mCRPC as compared to PCA, indicating a potential role of the ERα–NEAT1 interaction in the promotion of castration resistance. In PCA-patients, elevated NEAT1 levels are independently associated with early biochemical recurrence and metastatic spread [79]. Therefore, NEAT1 does not only constitute a potential target for PCA treatment, but may also serve as a reliable prognostic biomarker indicating early biochemical recurrence.

#### 3.3.4. Prostate Cancer-Associated Transcript 5

Fusions of ETS family transcription factors (e.g., ERG, ETV4) with regulatory sequences of androgen-regulated genes are found in over 50% of PCA patients [3]. By activating repressive epigenetic programmes via EZH2, ERG overexpression reduces AR expression and thus promotes androgen-resistance [81]. 

The lncRNA prostate cancer-associated transcript 5 (PCAT5) is particularly overexpressed in ERG-positive mCRPC compared to healthy prostate tissue (Figure 1C) [82]. Knockdown of either ERG or ETV4 leads to a distinct reduction in PCAT5-levels, indicating a direct regulation of this lncRNA by ETS family members [82]. In PC-3 cell lines, the knockdown of PCAT5 leads to enhanced apoptosis whilst reducing proliferation and invasion. Moreover, PC-3 cells lose the ability to form colonies and to migrate upon PCAT5-knockdown [82]. Thus, PCAT5 seems to be involved in the regulation of signalling pathways downstream of ERG [82]. In clinical practice, PCAT5 could therefore serve as a therapeutic target in ERG-positive mCRPC.

#### 3.3.5. Second Chromosome Locus Associated with Prostate-1

As with PCAT5, the lncRNA Second chromosome locus associated with prostate-1 (SChLAP1) is associated with ETS-positive PCA types and is overexpressed in about one-quarter of all PCA. Elevated SChLAP1-levels are even more frequent in mCRPC [83]. It promotes cancer cell invasion as well as metastatic spread and interacts with the SWItch/Sucrose Non-Fermentable (SWI/SNF)-complex. Any loss of this complex, that usually moves nucleosomes at gene promoters, results in cancer progression (Figure 1C) [84]. At the post-transcriptional level, SChLAP1 counteracts tumour-suppressive effects of the SWI/SNF complex by impairing its capability to regulate gene expression [83].

SChLAP1 levels do not only predict early biochemical recurrence in localised PCA [85], but also correlate with a highly aggressive disease process in mCRPC [86]. In particular, in patients with radical prostatectomy, SChLAP1 overexpression is independently associated with lethal mCRPC, irrespective of Gleason score, PTEN status, patient’s age and pathologic stage [86]. Therefore, this lncRNA could serve as a reliable prognostic biomarker, prompting early aggressive treatment as soon as patients with high SChLAP1-levels show evidence of biochemical recurrence.

#### 3.3.6. Suppressor of Cytokine Signalling 2-Antisense Transcript 1

The lncRNA Suppressor of cytokine signalling 2-antisense transcript 1 (LncRNA SOCS2-AS1) is located at the opposite strand of the protein coding gene region for SOCS2 [87]. Through a negative feedback mechanism, the suppressor of cytokine signalling (SOCS) protein family impedes further cytokine stimulation by reducing phosphorylation of proteins of the JAK/STAT (Janus Kinase/Signal Transducer and Activator of Transcription) signalling pathway [88]. Expression of both the protein SOCS2 and the lncRNA SOCS2-AS1 are regulated by androgens through androgen-responsive binding sites at the respective promoter regions [87].

Likewise, in the hormone-sensitive cell line LNCaP and castration-resistant cell line LTDA, the knockdown of SOCS2-AS1 and SOCS2 diminishes cell proliferation [87]. Conversely, overexpression of SOCS2-AS1 results in enhanced proliferation and migration of prostate cancer cells. On a molecular level, this lncRNA regulates genes involved in cell cycle, proliferation and apoptosis. For example, the tumour necrosis factor superfamily member 10 (*TNFSF10*), a gene downregulated most by SOCS2-AS1, belongs to pro-apoptotic protein ligands of the tumour necrosis factor superfamily [89].

Particularly in castration-resistant cell lines, knockdown of SOCS2-AS1 markedly induces expression of TNFSF10 and other apoptosis-related genes [87]. Consequently, high SOCS2-AS1 levels as present in mCRPC possibly contribute to tumourigenesis by promoting anti-apoptotic capabilities of tumour cells (Figure 1C) [87]. Simultaneous blockage of this lncRNA could therefore enhance the efficacy of novel anti-androgens and chemotherapeutics used for mCRPC treatment.

## 4. Conclusions

Due to the prolonged and varied disease process, treatment of prostate cancer has to be planned individually for each patient. On the grounds of thorough basic medical research performed over the last few years, molecular mechanisms underlying the pathogenesis of prostate cancer have been gradually uncovered. The introduction of novel anti-androgens into clinical practice has markedly improved life expectancy of patients resistant to conventional anti-hormonal therapy. Treatment may be adapted upon detection of specific biomarkers such as the AR-V7 splice variant in mCRPC. LncRNAs are involved in all these stages of tumour progression. They may preserve androgen-related pathways upon androgen deprivation, promote the progress towards castration-resistant states or maintain cellular proliferation and invasion independent from androgens. Some lncRNAs are already—or may be in the future—used as diagnostic biomarkers. Distinct lncRNA expression patterns can be prognostic or predictive. As therapeutic targets, lncRNAs could likewise enhance efficacy of anti-tumour agents and aid deceleration of prostate cancer progression.

The expression of lncRNAs can be regulated by using the RNA-interference (RNAi) technology. In this method, short double-stranded RNAs (e.g., siRNA) induce a RISC (RNA Induced Silencing Complex) -mediated degradation of their target lncRNA [90]. Therefore, the RNAi technology could be used to effectively reduce expression levels of lncRNAs with tumourigenic potential. Another method is based on the usage of antisense oligonucleotides (ASOs) that are either short single-stranded RNAs or DNAs antisense to their target lncRNA [91]. Moreover, the use of small molecules can, for example, inhibit the interaction of HOTAIR with LSD1 and PRC2 [92,93]. The therapeutic usage of H19-regulated double-stranded DNA plasmid BC-819 has already been successfully tested in patients with bladder cancer [94]. However, most studies using lncRNAs as therapeutic targets were performed on cell cultures or animal models and only few studies involving human subjects have been carried out. Moreover, the exact function of many lncRNAs is still unknown, since they do not necessarily have only one target or function within a cell. In addition, the same lncRNA may exert different functions depending on the type of tumour. Therefore, using lncRNAs as therapeutic targets may entail unforeseeable side effects or dramatic adverse reactions. Nevertheless, the better the function of lncRNAs is understood, the more efficient and broader their field of therapeutic usage will be. Moreover, ongoing research will uncover further lncRNAs involved in the pathogenesis of prostate cancer, their molecular effects and potential implication on clinical practice.

## Figures and Tables

**Table 1:** Clinical usage of aberrantly expressed lncRNAs in prostate cancer.

LncRNA	Expression Pattern	Relevance
**Diagnostic Biomarker**		
*PCA3*	Overexpression	Predicts prostate cancer in combination with PSA
*MALAT-1*	Overexpression	More sensitive than PSA for initial diagnosis
**Prognostic/Predictive Biomarker**		
*HOTAIR*	Overexpression	Associated with resistance to enzalutamide
*MALAT-1*	Overexpression	Correlates with ADT-resistance
*SChLAP1*	Overexpression	Predicts lethal mCRPC
*LncRNA-ATB*	Overexpression	Correlates with unfavourable clinical features
*PCAT29*	Underexpression	Associated with early biochemical recurrence
*DRAIC*	Underexpression	
*NEAT1*	Overexpression	Predicts early biochemical recurrence
**Therapeutic Target**		
*LncRNA-ATB*	Overexpression	Blockage could slow down tumour progression
*PCA3*	Overexpression	Inhibition may retard progression of disease
*MEG3*	Underexpression	Induction of expression could decelerate tumour progression
*DANCR*	Overexpression	Metastatic spread prevented by blockage upon enzalutamide-treatment
*PlncRNA-1*	Overexpression	Blockage could help to slow down cancer progression
*GAS5*	Underexpression	Indirectly upregulated by mTOR (mechanistic target of rapamycin)-inhibitors
*PCGEM1*	Overexpression	Efficacy of ADT enhanced by blockage
*CTBP1-AS*	Overexpression	Blockage could reduce proliferation rate
*HOTAIR*	Overexpression	Efficacy of enzalutamide enhanced upon blockage
*PCAT5*	Overexpression	Inhibition could be effective, particularly in ERG-positive prostate cancers
*SOCS2-AS1*	Overexpression	Blockage may reverse anti-apoptotic abilities

DANCR: differentiation antagonising non-protein coding RNA; MEG3: lncRNA Maternally expressed gene 3; PCA3: prostate cancer antigen 3; DRAIC: Downregulated RNA in cancer; PCAT29: Prostate cancer-associated transcript 29; GAS5: growth arrest-specific 5; CTBP1-AS: C-terminal binding protein 1-antisense; PCGEM: prostate cancer gene expression marker 1; MALAT-1: metastasis-associated lung adenocarcinoma transcript-1; NEAT1: nuclear-enriched abundant transcript 1; PCAT5: prostate cancer-associated transcript 5; SChLAP1: Second chromosome locus associated with prostate-1; HOTAIR: HOX transcript antisense RNA; SOCS2-AS1: cytokine signalling 2-antisense transcript 1; PSA: prostate-specific antigen; ADT: androgen deprivation therapy; mCRPC: castration-resistant prostate cancer; ERG: ETS- (E-twenty-six) related gene; mTOR: mechanistic target of rapamycin.

## Abbreviations

ADT	Androgen deprivation therapy
AR	Androgen receptor
BCL4	B-cell lymphoma like-2 like protein 4
BPH	Benign prostatic hyperplasia
CKS2	Cyclin-dependent kinases regulatory subunit 2
CTBP1	C-terminal binding protein 1-antisense
CTBP1-AS	C-terminal binding protein 1-antisense
DAB2IP	Disabled homolog 2-interacting protein
DANCR	Differentiation antagonising non-protein coding RNA
DRAIC	Downregulated RNA in cancer
EMT	Epithelial- to -mesenchymal transition
ERG	ETS-(E-twenty-six) related gene
ERα	Oestrogen receptor αalpha
EZH2	Enhancer of zeste homolog
FOXA1	Forkhead box protein A1
GAS5	Growth arrest-specific 5
HDAC-Sin3A	Histone decarboxylase paired amphipathic helix protein Sin3a
hnRNP A1	Heterogeneous nuclear ribonucleoprotein A1
HOTAIR	HOX transcript antisense RNA
lncRNA	Long non-coding RNA
MALAT-1	Metastasis-associated lung adenocarcinoma transcript-1
mCRPC	Metastatic castration-resistant prostate cancer
MDM2	Mouse double minute 2 homolog.
MEG3	LncRNA Maternally expressed gene 3
NEAT-1	Nuclear-enriched abundant transcript 1
NKX3-1	Homeobox protein Nkx-3.1
PCA	Prostate adenocarcinoma
PCA3	Prostate cancer antigen 3
PCAT29	Prostate cancer-associated transcript 29
PCAT5	Prostate cancer-associated transcript 5
PCCGEM	Prostate cancer gene expression marker 1
PRUNE2	Prune homolog 2
PSA	Prostate Prostate-specific antigen
SChLAP1	Second chromosome locus associated with prostate-1
SMAD3	Mothers against decapentaplegic homolog 3
SOCS2-AS1	Cytokine signalling 2-antisense transcript 1
SWI/SFN	SWItch Sucrose Non-Fermentable complex
TIMP 2/3	Tissue inhibitor of metalloproteinase
TMEM48	Transmembrane Protein 48
TMPRSS2	Transmembrane Protease, Serine 2
TNSF10	Tumour necrosis factor superfamily member 10
U2AF65	U2 Small Nuclear RNA Auxillary Factor 2
ZEB1	Zinc-finger E-box binding homeobox 1
ZNF217	Zinc finger protein 217
